# Review on Polymers for Thermoelectric Applications 

**DOI:** 10.3390/ma7096701

**Published:** 2014-09-18

**Authors:** Mario Culebras, Clara M. Gómez, Andrés Cantarero

**Affiliations:** Materials Science Institute, University of Valencia, P.O. Box 22085, 46071 Valencia, Spain; E-Mails: mario.culebras@uv.es (M.C.); clara.gomez@uv.es (C.M.G.)

**Keywords:** intrinsically conducting polymers, thermoelectrics, nanocomposites

## Abstract

In this review, we report the state-of-the-art of polymers in thermoelectricity. Classically, a number of inorganic compounds have been considered as the best thermoelectric materials. Since the prediction of the improvement of the figure of merit by means of electronic confinement in 1993, it has been improved by a factor of 3–4. In the mean time, organic materials, in particular intrinsically conducting polymers, had been considered as competitors of classical thermoelectrics, since their figure of merit has been improved several orders of magnitude in the last few years. We review here the evolution of the figure of merit or the power factor during the last years, and the best candidates to compete with inorganic materials. We also outline the best polymers to substitute classical thermoelectric materials and the advantages they present in comparison with inorganic systems.

## 1. Introduction

The interest in thermoelectricity has increased in the last few years since new applications in energy conversion had been envisaged. In the past, thermoelectric devices were basically that based on the Peltier effect and used in cooling applications, as small fridges, electrical and electronic household cooling systems, or Peltier coolers for laboratory detectors. The Peltier effect consists of the appearance of a temperature difference in a heterojunction when an electrical current cross it (*i.e.*, a current produces a temperature gradient). Two new interesting applications are the basis of the renew interest: the use of thermoelectric modules combined with photovoltaics and energy harvesting, *i.e.*, the conversion of waste heat into electricity (energy recovery). These devices would be based on the Seebeck effect, which consists of producing electricity from a temperature difference, the mirror of the Peltier effect (a temperature gradient produces a current when crossing a heterojunction). The thermoelectric modules can be used in photovoltaics to keep the temperature of the solar cell in the region of maximum efficiency. It can also be used in a complementary device: during the day the solar cell supplies energy, while during the night the thermoelectric module supplies energy (energy harvesting). A more interesting application is the use of thermoelectric modules by themselves to produce electricity from waste heat, basically the energy spectrum which cannot be used in photovoltaic applications [1,2] in the infrared spectral region (IR). 

**Figure 1:**
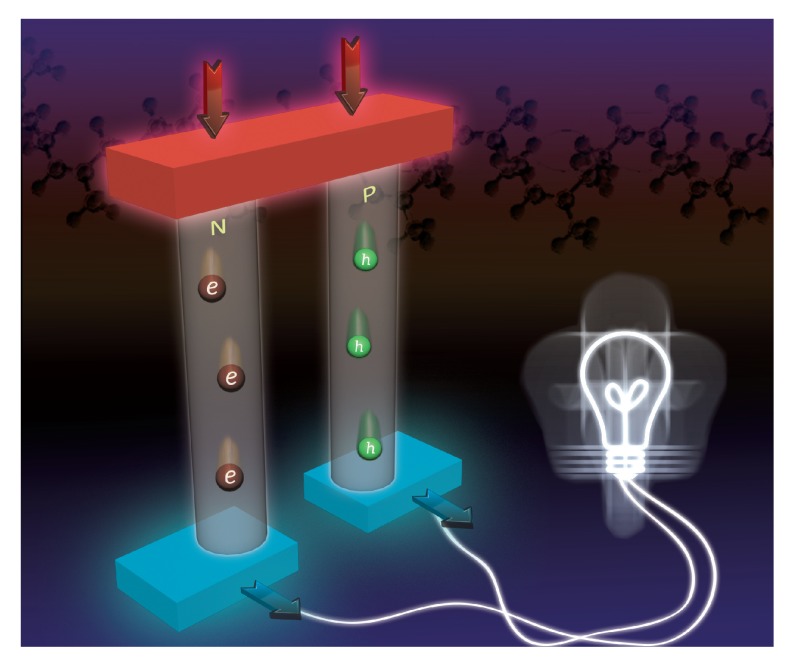
Thermoelectric module.

Figure 1 shows a single thermoelectric generator (TEG) based on a *p* − *n* junction working as a Seebeck current supply. The heat flow goes from the top to the bottom, while the current flows in the direction provided by the holes movement. However, in a Peltier module, since we are removing heat from the top, the temperature at the top is lower than that at the bottom. In the Seebeck module, the higher temperature at the top produces a current, *i.e.*, the temperature difference has a different sign that in the Peltier module. A real TEG consists of a series of modules as that shown in Figure 1 in serial. The TEGs have several attracting features as compared with, for instance, conventional turbines, engines or compressors: they do not have moving parts. They can be used to supply energy in pacemakers, defibrillators, or in cochlear implants and many other electronic devices which need a small amount of energy in a large time scale. Another advantage of a TEG is the high level of integration, very useful to remove heat in a network of electronic devices. 

The maximum thermoelectric efficiency of a thermoelectric generator is given by

(1) $$\phi_{max}=\frac{P_{out}}{Q_{in}}$$

where *Q_in_* is the amount of heat which enters into the device and *P_out_* is the power generated by the device, including the heat losses. In terms of the Carnot efficiency [3],

(2) $$\phi_{max}=\phi_{C}\frac{\sqrt{1+ZT_{av}}-1}{\sqrt{1+ZT_{av}}+T_{h}/T_{c}}\equiv \phi_{C}\gamma$$

*T_h_* and *T_c_* being the temperatures at the hot and cold ends, respectively, *T_av_* = (*T_h_* + *T_c_*)/2, and the Carnot efficiency is given by *ϕ_C_* = (*T_h_* − *T_c_*)/*T_h_*. The term *γ* represents the irreversible contribution to the efficiency. The quantity *ZT* is called figure of merit and it can be shown to be [3]

(3) $$ZT=\frac{\alpha^{2}\sigma }{\kappa }T$$

where *α* is the Seebeck coefficient; *σ* is the isothermal electrical conductivity; and *κ* the thermal conductivity. The thermal conductivity has actually two contributions, *κ* = *κ_e_* + *κ_p_*. The electronic contribution *κ_e_* is the most important in metals, while in semiconductors the phonon contribution *κ_p_* is dominating. 

**Figure 2:**
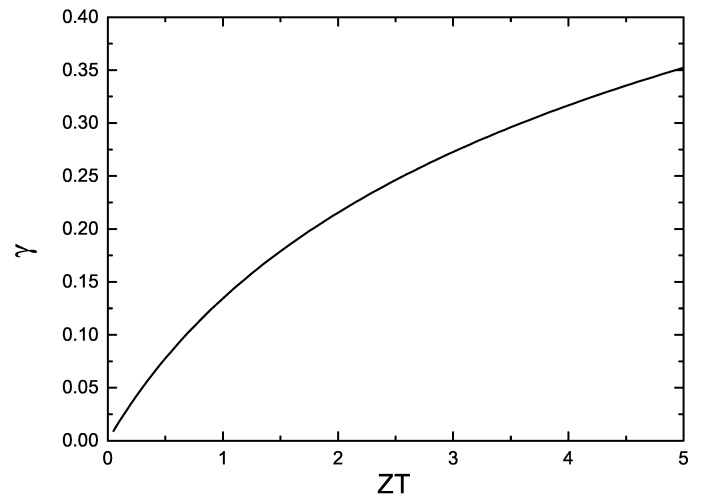
*γ* as a function of *ZT*.

From Equation (2), it is clear that the maximum efficiency depends on both the Carnot efficiency and *γ*. As an example, if the cold end is at room temperature and the hot end at 1000 K, *ϕ_C_* = 0.7 = 70%. In order to reach this efficiency, *γ* must be 1, which corresponds to *ZT* → ∞ (or *Z* → ∞). In Figure 2 we show the function *γ*(*ZT* ) from *ZT* = 0 to *ZT* = 5. Even in the case where *ZT* = 5 the value of *γ* = 0.42, *i.e.*, *ϕ_max_* = 28% following this example. With the state of the art in inorganic materials at present (*ZT* ∼ 2), the maximum efficiency would be *ϕ_max_* ∼ 20% in the present case. These are some limiting cases and depending on the application, not always such temperature gradients are possible. Large temperature gradients can be produced in the exhaust of a car. In a Peltier modulus to cool a detector the temperature difference is of the order of 40 K. In the case of organic materials, the best values of *ZT* are half of the values obtained in inorganic materials and the Carnot efficiency will be lower than 40%. Although the important factor to improve is not the figure of merit but the efficiency of the thermoelectric device, *i.e.*, *η_C_γ*, for a given application we can pay attention to the improvement of *ZT*. 

The electrical and heat current can be written, respectively, as

(4) $$\begin{array}{l} j=eL_{11}\left(eE-T\nabla \frac{µ}{T}\right)-eL_{21}\nabla \text{ln}T\\ j_{Q}=eL_{21}\left(eE-T\nabla \frac{µ}{T}\right)-L_{31}\nabla \text{ln}T \end{array}$$

where *µ* is the chemical potential (*µ* = *ε_F_*, the Fermi energy, at *T* = 0); ***E*** is the electric field and the transport coefficients are defined as [4]

(5) $$L_{ij}=\frac{4}{3m^{*}}\int \left(-\frac{\partial f}{\partial \varepsilon }\right)\tau^{j}\varepsilon^{i}D(\varepsilon )d\varepsilon$$

where m^*^ is the effective mass; *f* the distribution function (Fermi-Dirac); and *D*(*ε*) the electronic density of states. *τ*(*ε*) is the relaxation time, which depends on the scattering processes. In the absence of concentration and temperature gradients, the first of Equation (4) provides the electrical conductivity *σ* = *e*^2^*L*_11_. In an open circuit, *j* = 0 and we can deduced from Equation (4) that the electric field is

(6) $$E=\frac{1}{e}\nabla µ-\frac{µL_{11}-L_{21}}{eL_{11}T}\nabla T=-\nabla V$$

without gradients of concentrations we arrive to the expression of the Seebeck coefficient

(7) $$\alpha =\frac{µL_{11}-L_{21}}{eL_{11}T}=\frac{k_{B}}{e\sigma }\int_{\varepsilon_{c}}^{\infty }\sigma (\varepsilon )\left(\frac{\varepsilon -µ}{k_{B}T}\right)\left(-\frac{\partial f}{\partial \varepsilon }\right)d\varepsilon$$

this last equation was provided by Mott [5]. Since Equation (4) are only related to the electronic contribution, we can also deduce from them *κ_e_*. One way to derive an expression for the phononic part is via the Boltzmann transport equation for phonons [4]. Equation (7) is valid for insulators or metals. In the case of a metal or a degenerate semiconductor, the derivative of the distribution function is basically a Dirac delta function and the transport coefficients can be written as

(8) $$L_{ij}\approx \frac{4e^{2}}{3m^{*}}\tau^{j}(\varepsilon_{R})\varepsilon_{F}^{i}D(\varepsilon_{F})$$

thus the Seebeck coefficient given by Equation (7) can be written in the well known form [4,5]:

(9) $$\alpha =\frac{\pi^{2}}{3}{\frac{k_{B}^{2}T}{e}}^{\;}\frac{\partial \text{ln}\sigma }{\partial \varepsilon }{|}_{\;\varepsilon =\varepsilon_{F}}=\frac{\pi^{2}}{3}\frac{k_{B}^{2}T}{e\varepsilon_{F}}$$

This expression gives a Seebeck coefficient of the order of 1 µV·K^−^^1^. It is a typical value for metals. Actually, this expression, although using Fermi integrals, was used by Hicks and Dresselhaus [6] to claim that the figure of merit of Bi_2_Te_3_ could be increased up to a factor of 13 by reducing the dimensionality (building a superlattice). In practice, the figure of merit has been improved in the best of the cases by a factor of 3 by reducing the dimensionality [7]. The main goal to increase the figure of merit by decreasing the dimensionality is to engineer a material in such a way that the Fermi energy is just at the maximum of the density of electronic states. In this way, there will be a huge increase in the Seebeck coefficient, but of course there are other factors more difficult to engineer, mainly the thermal conductivity. 

The small thermal conductivity is actually one of the advantages of organic materials as compared to inorganic compounds. The thermal conductivity of most polymers is at least a factor of 10 smaller than that of inorganic compounds. In spite of the finding of Hicks and Dresselhaus [6], the figure of merit of inorganic compounds has increased by a factor of 3 or 4 in the last 20 years, and the increase was not exclusively due to the modification of the Seebeck coefficient, but to other facts related to the lattice thermal conductivity like interface roughness or, in general, to surface effects (see Ref. [8] and references therein). There are several more advantages in the use of conducting polymers instead of inorganic materials: the non scarcity of raw materials, the non toxicity, the possibility of using them in large area applications, *etc.* This last one is actually an important point, since as we commented previously the total efficiency depends on the Carnot efficiency multiplied by *ZT*. In large area applications we can, on one side, increase the temperature difference and, on the other, to increase the total supplied power by building a large area single module. One example of large area application can be found in the module proposed by Wagner *et al.* [9] using SiGe alloys, but here the Ge amount used in the module increases the cost of the device and it cannot be developed in practice. We have also developed a module for large area applications based on a p-type conducting polymer [10]. 

In the last years, most of the research in thermoelectricity has been concentrated in inorganic compounds. The main purpose was to find an inorganic material with a small lattice thermal conductivity. Besides the well known bismuth telluride, skutterudites [11] and half-Heusler compounds [12] have been studied in the last decade as good candidates for the development of TEGs. The strategy in the search of inorganic compounds with low thermal conductivity is to find materials with complex crystal structures and heavy atoms providing a low speed of sound (a complex dispersion relation), limiting the phonon transport. However, the real fact is that, while *ZT* in inorganic materials has increased in a factor of 3–4 in the last 20 years, even via nanostructuration, in the case of organic materials the increase in *ZT* has been of several orders of magnitude. Although 10 years ago the figure of merit of most conducting polymers were of the order of 10^−^^4^, nowadays the best values are around 0.5. In a recent work [10] we show a chemical route to further improve *ZT* and we expect in a few years similar values to that of inorganic systems. 

Table 1 shows the chemical formulas of conducting polymers commonly used in thermoelectricity: polyaniline (PANI), polyalkyl thiophenes, poly(3,4-ethylenedioxythiophene) (PEDOT), polyacetylene, polypyrrole (PPy) and poly(2,7-carbazolyenevinylene). In this review we will study the most important polymers and polymer nanocomposites used as TE materials and will analyze the evolution of the figure of merit along the last years. In Section 2, the most important works on conducting polymers, including the effect of doping and de-doping will be discussed. Section 3 reports on the improvement of the thermoelectric properties of polymers mixed with graphite/graphene, carbon nanotubes or inorganic thermoelectric nanoparticles and the few works using co-polymers for thermoelectric applications. In Section 4, the theoretical models used in the interpretation of the transport properties in polymers and polymer nanocomposites will be discussed. Finally, Section 5 gives some future perspectives on the thermoelectric properties of polymers. 

**Table 1:** Molecular structures of typical conductive polymers.

Polymer	Structure	Polymer	Structure
Polyacetylene	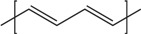	Polyaniline	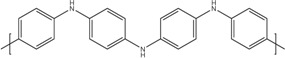
PEDOT	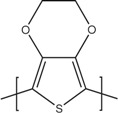	Polypyrrole	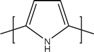
Polyalkyl thiophenes	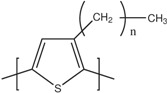	Poly(2-7carbazoles)	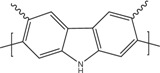

## 2. Effect of the Doping Level on the Thermoelectric Properties of Conductive Polymers

The thermoelectric performance is strongly dependent on the material doping level, due to inherent changes on the electrical conductivity and Seebeck coefficient [10,13,14,15,16]. The electrical conductivity increases as the doping level increases while the Seebeck coefficient decreases. For this reason, an optimal compromise between electrical conductivity and Seebeck coefficient should be reach to obtain the maximum thermoelectric efficiency (maximum power factor *PF* = *α*^2^*σ*). In the last years, many works dealing with the change of the doping level in conductive polymers have been published. Since this topic is of special interest for designing devices, several methods to control the doping level in conductive polymers will be analyzed in the next subsections. 

### 2.1. Chemical Doping and De-Doping

**Figure 3:**
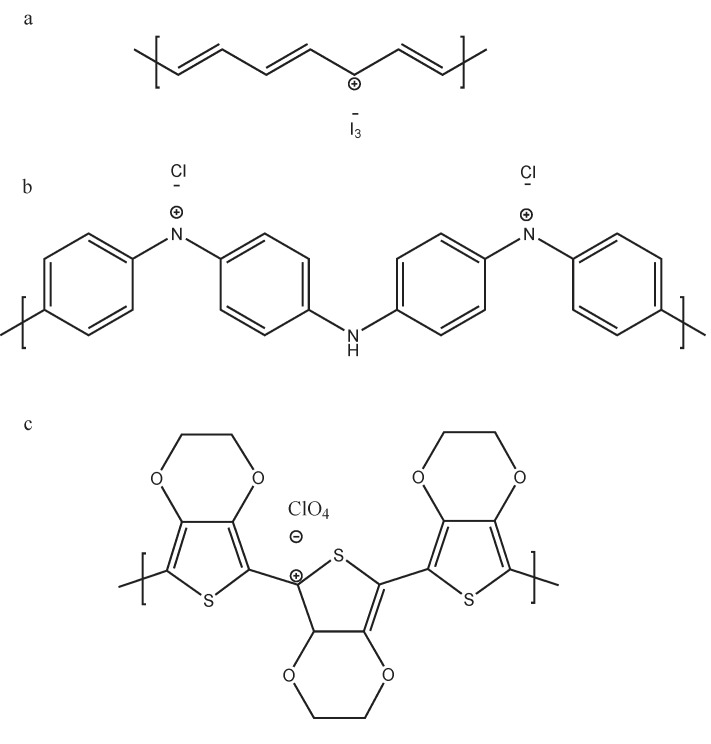
Doping the molecular structure of: (**a**) polyacetylene; (**b**) polyaniline (PANI) and (**c**) polyalkyl thiophenes, poly(3,4-ethylenedioxythiophene) (PEDOT).

The oxidative chemical polymerization is the most usual method to synthesize conductive polymers [15,16,17,18]. Basically, this method consists of the reaction between the monomer and an oxidative salt that has the role of a dopant agent. Typically, polymers synthesized by this method are p-type semiconductors, exhibiting an electron deficiency along its backbone. The positive charges generated (polaron-bipolaron states [19,20]) are neutralized by the dopant agent as shown in Figure 3. As the positive charges along the polymer backbone increase, the doping level increases. Table 2 shows the most usual dopant molecules of typical semiconducting polymers used in thermoelectric applications. 

**Table 2:** Dopants in the most typical semiconducting polymers.

Polymer	Dopant	References	Polymer	Dopant	References
PEDOT	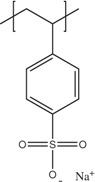	[13,14,21,22,23]	PANI	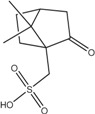	[24,25,26]
	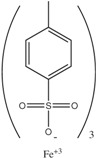	[14,15,16]		H_2_SO_4_	[27,28]
	LiClO_4_	[10,29,30]		HCl	[31]
	BF_4_	[32,33,34]		H_3_PO_4_	[35]
	PF_6_^−^	[10,29,36]	Polycarbazoles	FeCl_3_	[37]
Polyalkyl thiophenes	FeCl_3_	[38]	Polyacetylene	I_2_ vapour	[39,40]
	I_2_ vapour	[38]			

The first semiconducting polymer synthesized was the polyacetylene, in the decade of the seventies [41]. This polymer, doped with iodine vapour, has a very high electric conductivity, from 1 − 3 × 10^4^ S·cm^−^^1^ [39]. However, its low Seebeck coefficient and high thermal conductivity, depending on iodine doping level [40], makes polyacetylene to depict a low thermoelectric efficiency (*PF* = 8.3 × 10^−^^5^ − 2 × 10^−^^3^ W·m^−^^1^·K^−^^2^). 

In the case of PANI, the doping level can be controlled with the molar ratio of the acid used in its synthesis, as previously reported [24,31,35]. The electrical conductivity could be controlled in the range from 1 to 6 S·cm^−^^1^, depending on the concentration of HCl. The electrical conductivity increases as the HCl concentration increases. However, the Seebeck coefficient shows an opposite trend: at low HCl concentration it increases up to 35 µV·K^−^^1^ [31]. 

**Figure 4:**
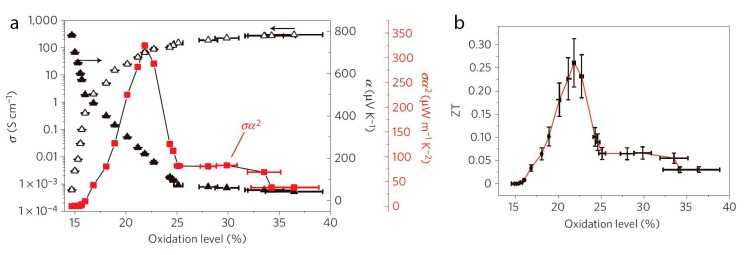
(**a**) Seebeck coefficient (filled triangles), electrical conductivity (open triangles), corresponding power factor (red squares); and (**b**) *ZT* values of PEDOT:Tos *versus* oxidation level at room temperature [15]. Reproduced with permission of Nature Materials.

The *ZT* values of conductive polymers were established around 10^−^^3^ until 2011, when Bubnova *et al.* [15] reported the optimization of *ZT* in PEDOT:p-toluenesulfonate (PEDOT:Tos) by using tetrakis(dimethylamino)ethylene (TDAE) as de-doping agent. When a PEDOT:Tos film with a high doping level (high oxidation level) is subjected to TDEA vapour, the electron deficiency on the PEDOT:Tos backbone is neutralized, the number of charge carriers decreases and a de-doping process occurs. The electrical conductivity decreases and the Seebeck coefficient increases as shown in Figure 4. At 22% of oxidation level in PEDOT:Tos, a very high power factor of 324 µW·m^−^^1^·K^−^^2^ was obtained. By assuming a value of *κ* ≈ 0.35 W·m^−^^1^·K^−^^1^, *ZT* ≈ 0.25. 

### 2.2. Electrochemical Doping/De-Doping

**Figure 5:**
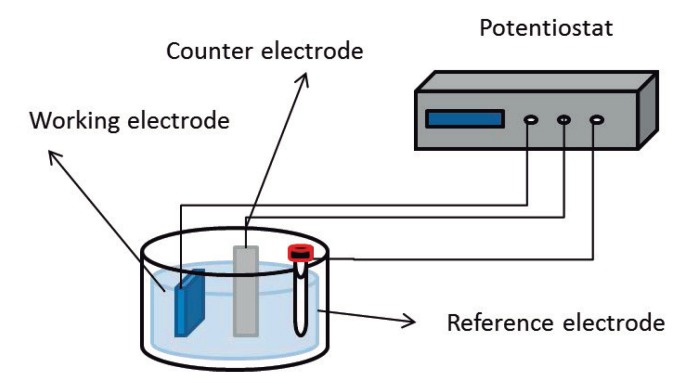
Scheme of the electrochemical cell.

The electrochemical synthesis is a suitable method for the preparation of conductive polymers, such as PPy, PEDOT and PANI, as reported in the literature [10,42,43]. The polymer doping level can be easily controlled by using an electrochemical cell. The electrochemical de-doping/doping process is carried out in a three electrode cell with an electrolyte solution as shown in Figure 5. The conductive polymer is the working electrode where the oxidation/reduction process occurs, generally a platinum wire is the counter electrode and Ag/AgCl is the reference electrode. 

**Figure 6:**
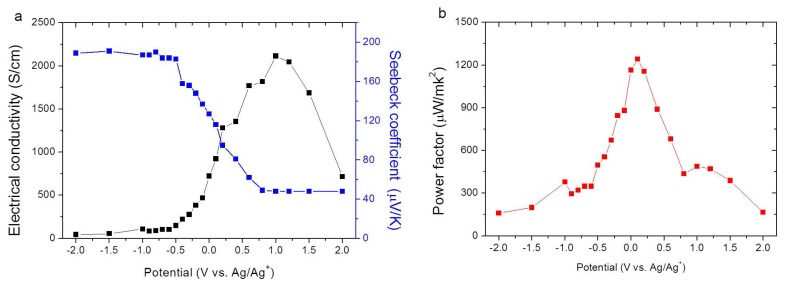
(**a**) Electrical conductivity, Seebeck coefficient; and (**b**) power factor of PEDOT:Tos as a function of potential (V *vs.* Ag/Ag^+^) [16]. Reproduced with permission of Energy and Environmental Science.

PEDOT:Tos synthesized by oxidative polymerization in the presence of Poly(ethylene glycol)-block-poly(propylene glycol)-blockpoly(ethylene glycol) triblock copolymer (PEO-PPO-PEO) [16] has yielded the highest *PF* value obtained until now. As shown in Figure 6a the electrical conductivity increases as the Seebeck coefficient decreases when the applied potential changes from 0 to 1.1 V. The best power factor of 1270 µW· m^−^^1^·K^−^^2^ is obtained at 0.1 V as depicted in Figure 6b. 

**Figure 7:**
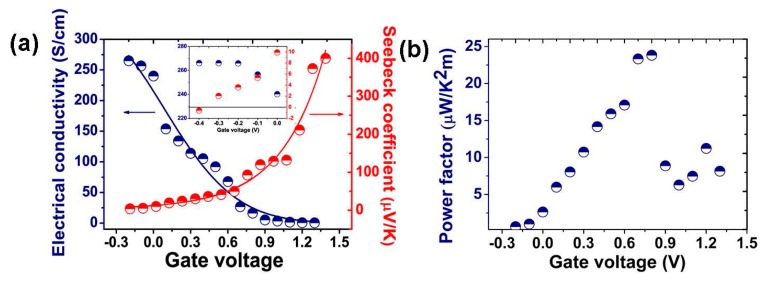
(a) Electrical conductivity and Seebeck coefficient; (b) power factor of PEDOT:PSS as a function of gate voltage [21]. Reproduced with permission of the Journal of the American Chemical Society.

An organic electrochemical transistor to control the oxidation state (doping level) of PEDOT:PSS has been reported [21]. The electrical conductivity, Seebeck coefficient and power factor were determined as a function of gate voltage as shown in Figure 7. The best power factor obtained was 23.5 µW·m^−^^1^·K^−^^2^ that corresponds to *ZT* = 0.041, using a value of *κ* ≈ 0.17 W·m^−^^1^·K^−^^1^ . 

### 2.3. Secondary Doping

**Figure 8:**
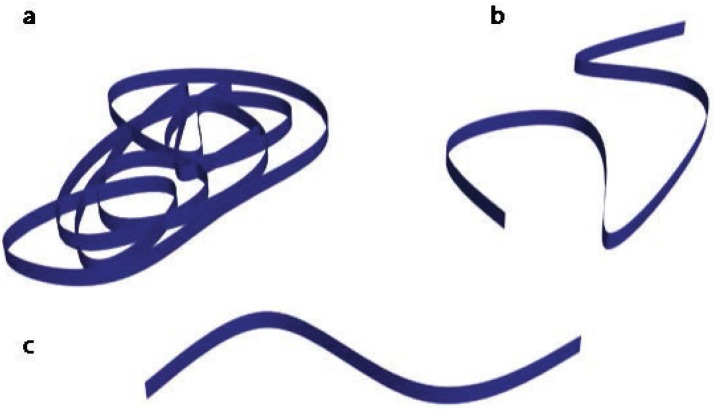
(**a**) Coil conformation; (**b**) extended coil conformation; and (**c**) linear conformation.

The other kind of doping polymers is called secondary doping. This doping is not electronic, it is related to the polymer chain conformation. The polymer conformation may play an important role in its physical properties, in particular in the conductivity. The electron-hole interaction leads to strongly bonded excitons (Frenkel excitons), which greatly affects the transport properties in conducting polymers. The electronic transport is traditionally explained by the charge-energy-limited-tunneling model, proposed originally for highly disordered conducting polymers [22,23] (for low electric fields, the voltage and the current are not proportional, since there is carrier injection). Amorphous polymer chains adopt a random coil conformation. The electrical conductivity of a random coil is lower than that of an extended coil/linear conformation (see Figure 8). Thus, the control of the polymer conformation is crucial to improve the electrical conductivity in order to obtain a high thermoelectric efficiency. 

In the case of PEDOT:PSS, addition of organic solvents such as dimethyl sulfoxide (DMSO) or ethylene glycol (EG) would improve the electrical conductivity by several orders of magnitude. This effect is due to the resonant structure of the PEDOT chain that changes from a benzoid to a quinoid structure, due to Van der Waals interactions between PEDOT chains and the organic solvent. 

Many papers have reported the thermoelectric behaviour of PEDOT:PSS with DMSO or EG [23,44,45,46]. Overall, the electric conductivity of PEDOT:PSS without additives is around 10^−^^1^ S·cm^−^^1^. Addition of DMSO increases the conductivity up to 200–900 S·cm^−^^1^, depending on the PEDOT:PSS ratio. The Seebeck coefficient remains constant, around 12–20 µV·K^−^^1^, for both EG or DMSO content. The figure of merit ZT increases from 10^−^^4^ to 10^−^^2^ with the addition of 5%–10% of organic additive [23,44,46,47]. 

**Figure 9:**
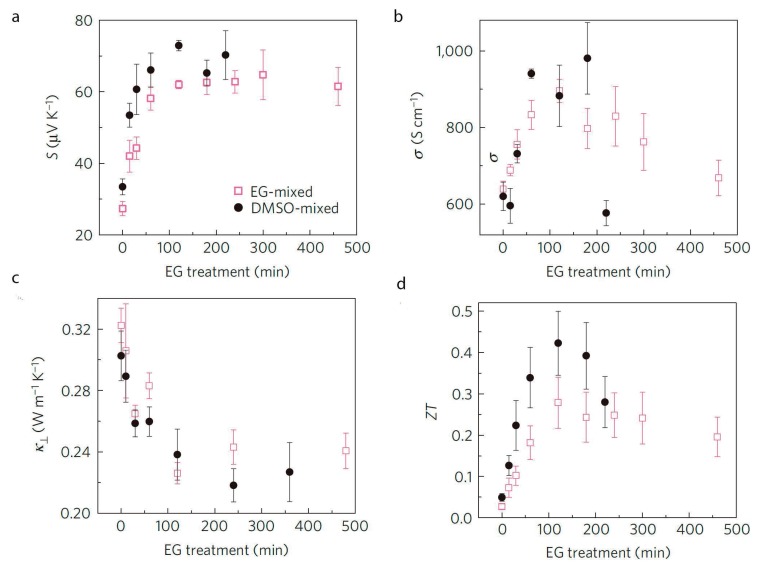
(**a**) Seebeck coefficient; (**b**) electrical conductivity; (**c**) thermal conductivity and (**d**) ZT of PEDOT:PSS as a function of ethylene glycol (EG) treatment [48]. Reproduced with permission of Nature Materials

PEDOT:PSS is formed by two kinds of polymers: PEDOT, the conductive polymer and PSS, an insulating polymer. Generally, there is an excess of PSS in the PEDOT:PSS solution. A recent work published by Pipe *et al.* [48] reported the thermoelectric measurements of PEDOT:PSS with 5% of DMSO and EG after submerging the films in EG several times (from 0 to 450 min) in order to remove the PSS in excess. The insulating polymer (PSS) is removed and, consequently, the electrical conductivity and Seebeck coefficient increases simultaneously as shown in Figure 9. The *ZT* value of 0.40 reported in this work is the highest ever obtained for a polymer until date. 

**Figure 10:**
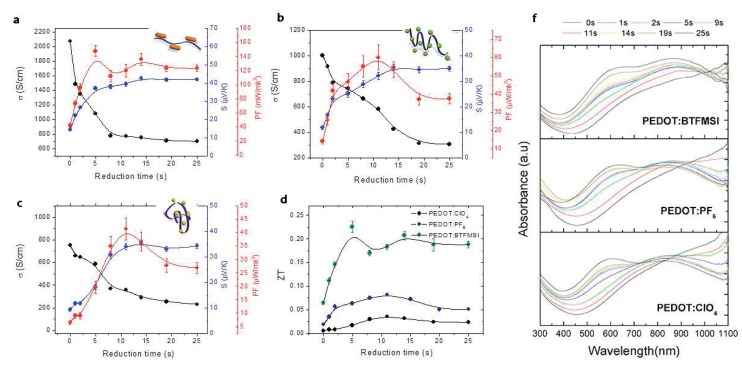
Electrical conductivity and Seebeck coefficient of: (**a**) PEDOT:BTFMSI; (**b**) PEDOT:PF_6_; (**c**) PEDOT:ClO_4_; (**d**) *ZT* and (**e**) UV-Vis-NIR spectra of the three polymers, as a function of the reduction time. Reproduced with permission of the Journal of Materials Chemistry A.

In the electrochemical synthesis of PEDOT films, the counter-ion used as a dopant may have an important role in the polymer chain conformation as shown in Figure 8 . In a previous work [10], Culebras *et al.* reported the thermoelectric measurements of PEDOT synthesized in the presence of three different counter-ions: ClO_4_, PF_6_ and bis(trifluoromethylsulfonyl)imide (BTFMSI). Figure 10 shows the evolution of the electrical conductivity, Seebeck coefficient, *PF* and *ZT* of the three polymers as a function of the reduction time. Maximum values of 753 S·cm^−^^1^ for PEDOT:ClO_4_, 1000 S·cm^−^^1^ for PEDOT:PF_6_ and 2074 S·cm^−^^1^ for PEDOT:BTFMSI have been determined. A change from a typical coil conformation to a linear or expanded-coil conformation (see Figure 8) takes place in the presence of the different counter-ions or dopants. The evolution of the chain conformation with the size of the counter-ion increases the compactness of the films, thus increasing the electrical conductivity. In addition, the thermoelectric efficiency was optimized by chemical reduction with hydrazine at different times as shown in Figure 10. The combination of secondary doping with chemical de-doping allows an increase in *ZT* until 0.22 [10]. Figure 10f indicates the UV-Vis-NIR absorption as a function of the reduction time. A broad absorption band centered around 600 nm is observed at negative potentials that correspond to the *π*−π^*^ transition, due to neutral segments in PEDOT chains (low doping). The other band around 900 nm is related to the doping process of PEDOT, associated to bipolaron states [19,20] (high doping level). 

**Figure 11:**
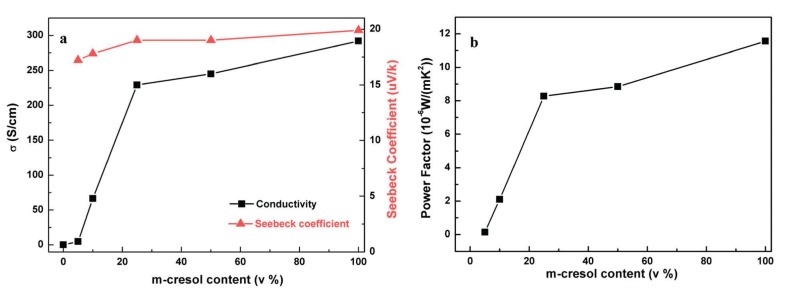
(**a**) Electrical conductivity and Seebeck coefficient (**b**) and power factor of polyaniline as a function of m-cresol content [25]. Reproduced with permission of the Journal of Materials Chemistry A.

In the case of PANI, it is possible to produce a secondary doping with m-cresol [25,26]. The addition of m-cresol to a PANI chloroform solution (doped with CSA), generates an increase of the electrical conductivity from 10^−^^1^ to 10^2^ S·cm^−^^1^ . A solution of PANI molecules show a compacted coil conformation due to strong van der Waals interactions. The addition of m-cresol to CSA-doping PANI generates additional interactions between the carbonyl group of CSA and the hydroxyl group of m-cresol. This hydrogen bonding alters the ionic bond between the positive imine ions and negative CSA ions. The electrostatic repulsion increases, are higher than the van der Waals interactions, thus changing the conformation from compacted to expanded coil [25,26]. The thermoelectric properties as a function of m-cresol content of a PANI chloroform solution were reported by Qin Yao *et al.* [25]. The electrical conductivity and Seebeck coefficient increase with m-cresol content as shown in Figure 11. The power factor was around 10 µW·m^−^^1^·K^−^^2^, denoting a big increase compared with previous results without m-cresol [17,24,49]. 

### 2.4. Effect of pH on the Thermoelectric Properties of PEDOT:PSS

The most employed conductive polymer in electronic applications is PEDOT:PSS, due to its inherent properties such as solubility in water. The pH of a PEDOT:PSS/water solution affects the thermoelectric properties as it has been previously reported [50,51]. The addition of NaOH produces the neutralization of the PSS anions as shown in the reaction:

(10) PSS^−^H^+^ + Na^+^OH^−^→ H_2_O + Na^+^PSS^−^

As the pH increases, the PSS doping concentration is reduced, the electrical conductivity decreases and the Seebeck coefficient increases. Tsai *et al.* [50] reported values of the electrical conductivity and Seebeck coefficient for a 5 wt% DMSO PEDOT:PSS solution with pH from 1.15 to 13.42. The electrical conductivity changes from 900 to 250 S·cm^−^^1^ and the Seebeck coefficient from 10 to 27 µV·K^−^^1^ . The maximum value of *PF* = 19.6 µW·m^−^^1^·K^−^^2^ was obtained for a pH of 1.8. 

### 2.5. Co-polymers and Polymer Blends

**Figure 12:**
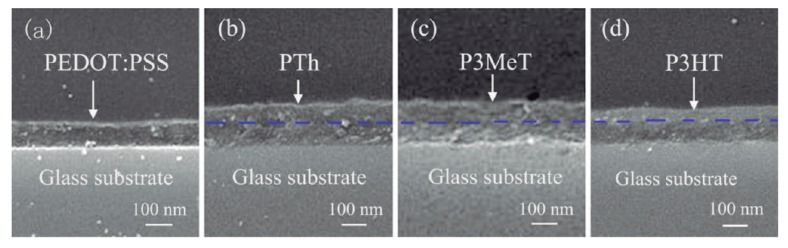
Cross-section scanning electron microscope (SEM) images of the nanofilms: (**a**) PEDOT:PSS; (**b**) PEDOT:PSS/PTh; (**c**) PEDOT:PSS/P3MeT; (**d**) PEDOT:PSS/P3HT [53]. Reproduced with permission of ACS Applied Materials and Interfaces.

Molecular engineering of the polymer chains could be the key to improve the thermoelectric efficiency of polymers in the near future [38,45,52]. On one hand, the co-polymerization is a strong tool to incorporate molecular segments of different nature in a polymer chain. This method allows to synthesize co-polymers with better thermoelectric efficiency than the corresponding homo-polymers. In addition, by using co-polymerization, the mechanical and chemical properties can be tailored. Until date, very promising values of *PF* have been obtained for, oligophenylenevinylene segmented block copolymers and their blends with MEH-PPV, 1.33 µW·m^−^^1^·K^−^^2^ [52], polyselenophene and its copolymers with 3-methylthiophene via electropolymerization, 12 µW·m^−^^1^·K^−^^2^ and a copolymer of 1,12-bis(carbazolyl)dodecane and thieno[3,2-b]thiophene, 0.32 µW·m^−^^1^·K^−^^2^ . On the other hand, by multilayer structuring, polymer films made with different layers of conductive polymers yield exciting results. Hui Shi *et al.* [53] reported the thermoelectric properties of PEDOT:PSS/Polythiophenes bilayered nanofilms (Figure 12). The best *PF* (6.0 µW·m^−^^1^·K^−^^2^) was obtained for a PEDOT:PSS/P3HT bilayered nanofilm. 

## 3. Improvement of the Figure of Merit Using Polymer Composites

Nowadays, most of the materials surrounding us are made from mixtures or blends of different components with the purpose of improving the physical properties by the synergy of properties of the raw components of the mixture. In this direction, we find polymer blends built up from a conducting polymer with highly conductive nanoparticles in order to increase the figure of merit. Overall, two kinds of materials can be used to improve the thermoelectric efficiency of a polymer matrix. On one hand inorganic semiconductors with high electrical conductivity such as carbon nanotubes, and on the other inorganic materials with high Seebeck coefficient. It is expected that the final composite will maintain low thermal conductivity, good processability and other related properties intrinsic to the polymer matrix like mechanical flexibility, low-cost synthesis, and light weight. The percolation threshold has to be reached to obtain a significant improvement of the matrix properties. Above a certain threshold, connection of adjacent particles increases the mean free path thus increasing the conductivity. The use of nanostructures allows the selective blocking of phonons whilst allowing the transport of charge carriers. 

### 3.1. Carbon Nanotubes

Carbon nanotubes are allotropes of carbon with a cylindrical nanostructure. Due to their extraordinary mechanical and electrical properties they are being extensively used as additives for polymer matrices. Multiwall (MWCNT), singlewall (SWCNT) or doublewall carbon nanotubes (DWCNT), functionalized or not, are being extensively used to improve electrical conductivity and mechanical properties of polymeric matrices [54]. 

**Figure 13:**
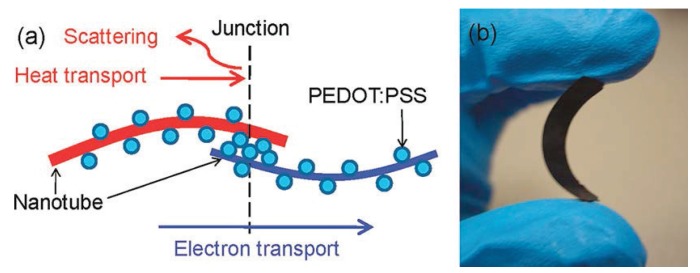
(**a**) Nanotubes are coated by PEDOT:PSS particles, making nanotube-PEDOT:PSS-nanotube junctions in the composites; (**b**) fully dried composite held between two fingers indicates that it is a free-standing flexible black material. Reproduced with permission of ACS Nano.

Choongho *et al.* have devoted several papers [55,56,57,58] to investigate the influence of carbon nanotubes on the thermoelectric performance of a polymer matrix. They started by studying composites of carbon nanotube (CNT) and poly(vinyl acetate) (PVAc) from aqueous solutions [55]. The highest thermoelectric performance was attained at a 20 wt% content of CNTs, with an electrical conductivity of 48 S·cm^−^^1^, thermal conductivity of 0.34 W·m^−^^1^·K^−^^1^ and a thermoelectric figure of merit larger than 6 × 10^−^^3^ at room temperature. Lately, by using a conductive polymer matrix, PEDOT:PSS doped with DMSO, in a SWCNTs dispersion they obtained promising ZT values around 0.02 [56]. The addition of 35 wt% of SWCNT to PEDOT:PSS give values of σ increasing up to 400 S·cm^−^^1^, while keeping constant *α* ≈ 20 µV·K^−^^1^, and the thermal conductivity *κ* ∼ 0.2 − 0.4 W·m^−^^1^·K^−^^1^ [56]. The addition of polyvinyl acetate to single-wall carbon nanotubes and PEDOT:PSS gives high electrical conductivities of the order of 10^3^ S·cm^−^^1^, keeping constant the thermal conductivity, *κ* ∼ 0.2 − 0.4 W·m^−^^1^·K^−^^1^, the thermopower *α* = 41 µV·K^−^^1^ and large thermoelectric power factors of about *PF* = 160 µW·m^−^^1^·K^−^^2^ with a 60 wt % SWCNT. The presence of PVAc in the composites improves the CNTs dispersion in the polymer matrix (Figure 13), thus enhancing the electrically connected junctions in the nanotube network and yielding high thermoelectric performance [57]. Recently, the addition of conductive stabilizer such as meso-tetra(4-carboxyphenyl) porphine (TCPP) to the PEDOT:PSS/DWCNTs dispersion clearly improves the thermoelectric properties of the composite giving very exciting values of *PF* = 500 µW·m^−^^1^·K^−^^2^, one of highest obtained for an organic flexible material [58]. 

**Figure 14:**
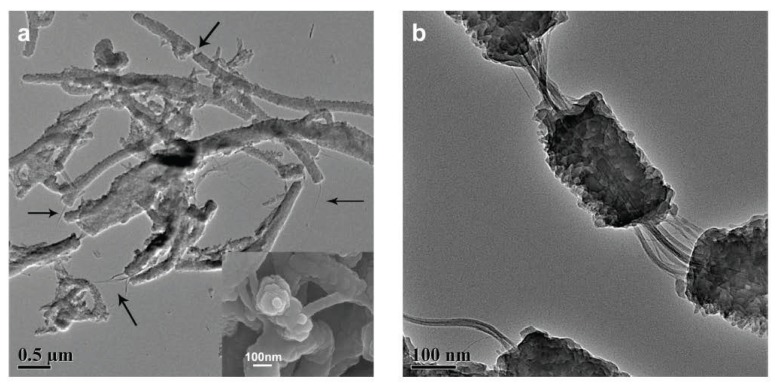
TEM images for singlewall carbon nanotubes (SWCNT)/PANI composites with 25 wt % SWCNT. Inset of (**a**) is the SEM top view of the nanocable. Reproduced with permission from ACS.

Generally, the power factor of most of the polymers used as thermoelectric materials is in the range of 1 − 10^−^^4^ µW·m^−^^1^·K^−^^2^, three orders of magnitude smaller than that the state-of-the-art in inorganic TE materials [59]. Addition of CNTs creates an interconnected network and increases the electrical conductivity, keeping nearly constant the Seebeck coefficient and the thermal conductivity. Building highly ordered structures will improve thermoelectric efficiency, as shown in composites formed by novel 3D CNT networks with PANI [60]. These composites depict maximum values of *σ* = 40.35 S·cm^−^^1^ , *α* = 23.3 µW·K^−^^1^ , *κ* = 0.29 W·m^−^^1^·K^−^^1^ that gives a ZT value of 2.2 × 10^−^^3^ . Notice that the *ZT* values are 6.0 × 10^−^^4^ for a 3D CNT network and 7.0 × 10^−^^7^ for PANI. Better results have been obtained for the case of SWCNTs dispersed in an aniline solution and in situ polymerization (Figure 14) [59]. 

The SWCNT/PANI nanocomposites show both higher electrical conductivity and Seebeck coefficient as compared to pure PANI, which could be attributed to the enhanced carrier mobility in the ordered chain structures of the PANI. The maximum electrical conductivity and Seebeck coefficient of composites reach 1.25 × 10^2^ S·cm^−^^1^ and 40 µW·K^−^^1^, respectively, and the maximum power factor is up to 2 × 10^−^^5^ W·m^−^^1^·K^−^^2^, more than 2 orders of magnitude higher than pure polyaniline, and *ZT* ∼ 0.004 [59]. The SWCNT/PANI nanocomposites show both higher electrical conductivity and Seebeck coefficient as compared to pure PANI, which could be attributed to the enhanced carrier mobility in the ordered chain structures of the PANI. 

The incorporation of SWCNTs to polythiophenes derivatives, especially poly(3-hexylthiophene), has demonstrated a high increase in their thermoelectric properties [61]. The power factor significantly exceeds the values obtained with either constituent alone, provided that the conjugated polymer is sufficiently p-doped. The use of SWCNTs consistently results in a higher electrical conductivity, with a maximum value above 1000 S·cm^−^^1^ and thus gives rise to a power factor of 25 µW·m^−^^1^·K^−^^2^ for a filler content of only 8 wt% and a maximum value of *PF* = 95 µW·m^−^^1^·K^−^^2^ for 42–81 wt%. Moreover, a CNT content of 8–10 wt% does not compromise the low bulk thermal conductivity of the polymer matrix, which promises a high figure of merit of at least *ZT* > 0.2 at room temperature. 

### 3.2. Graphene/Graphite

Starting from 2013, a lot of research has been devoted on how to increase the thermolectric properties by composite engineering with carbon derivatives different from CNTs-based materials as graphene (GN), graphene nanoplateletes (GNP) or expanded graphite. Graphite in all its variants has been extensively studied [62] as it is used to increase the electrical conductivity of polymer matrices. Different groups have attempted to improve the thermoelectric efficiency or turning from p-type to n-type conduction a PANI matrix [63,64,65,66]. Lei Wang *et* *al.* [63] prepared HClO4-doped polyaniline/graphite composites by mechanical ball milling and cold pressing. The Seebeck coefficient as well as the electrical conductivity increases with the graphite content giving a *PF* = 4.18 µW·m^−^^1^·K^−^^2^ and a figure of merit *ZT* = 1.37 × 10^−^^3^ at 50 wt% of graphite. More recently [65], similar values have been obtained for the same system, *PF* = 0.8 µW·m^−^^1^· K^−^^2^ and *ZT* =1.95 × 10^−^^3^ for a composite containing 30 wt% GN at 453 K. Simultaneous increase in electrical conductivity and Seebeck coefficient in PANI/graphene nanosheets nanocomposites has been obtained [64] for composites prepared as pellets and films. The power factor of the pellets and films increase from 0.64 to 5.60 and from 0.05 to 1.47 µW·m^−^^1^·K^−^^2^ at 50 wt% GN. The best value of PF = 14 µW·m^−^^1^·K^−^^2^ [66] has been determined for exfoliated graphene nanoplatelets/PANI composites at 50 wt% GNP by mechanical blending. 

A very promising paper reported values of *PF* = 11.09 µW·m^−^^1^·K^−^^2^ and *ZT* = 2.1 × 10^−^^2^ measured on spin coating films obtained by incorporating 2 wt% of graphene to a solution of PEDOT:PSS [67]. The uniformly distributed graphene increases the interfacial area by 2–3 times with respect similar CNT samples. This results in a facilitated carrier transfer between PEDOT:PSS and graphene as well as the high electron mobility of graphene. Additionally, the porous structure of the thin film decreases the thermal conductivity [67]. Films of PEDOT:PSS/expanded graphite [68] depict an exponential increase of the electrical conductivity while the Seebeck coefficient remains constant, with a maximum *PF* = 5.31 µW·m^−^^1^·K^−^^2^. In order to enhance the thermoelectric properties, non-covalently functionalized graphene with fullerene [69] by *π* − *π* stacking in a liquid interface was integrated into PEDOT:PSS. Graphene helps to improve the electrical conductivity while fullerene enhances the Seebeck coefficient and hinders the thermal conductivity. The electrical conductivity increases up to 700 S·cm^−^^1^, the thermal conductivity changed from 0.2 to 2 W·m^−^^1^·K^−^^1^, as usual in this kind of composites, while the Seebeck coefficient was enhanced by around 4-fold, yielding a *ZT* = 6.7 × 10^−^^2^ for 30 wt% nanohybrids-filled polymer composite where the ratio of fullerene to graphene was 3:7. Expanded graphite serves as a filler for both p-and n-type organic materials for constructing thermoelectric devices [70]. PEDOT:PSS/expanded graphite composites behave as p-type materials, as reported, while Expanded graphite dispersed in polyvinyl alcohol (PVA) with PEI gives composite films with improved n-type characteristics. Promising values of *α* = −25 µV·K^−^^1^ and *σ* = 10 S·cm^−^^1^ have been measured. 

### 3.3. Inorganic Nanoparticles

Several pioneering works [71,72,73] studied the influence of some inorganic structures on the thermoelectric efficiency of polymer matrices. PPy-Fe_2_O_3_ composites depict a linear metallic behavior of the Seebeck coefficient with positive values increasing with temperature and a semiconducting-like behavior of conductivity [71]. Thermoelectric hybrid materials of Bi_0__.__5_*_S_*b_1__.__5_Te_3_ and 1–7 wt %PANI [72] depict electrical conductivities and Seebeck coefficient 30%–70% lower than the corresponding to Bi_0__.__5_*_S_*b_1__.__5_Te_3_ sample implying a decreases of the *PF* from 250 to 90 µW·m^−^^1^·K^−^^2^ upon addition of 7 wt% of PANI. PANI/NaFe_4_P_12_ whisker and nanowire composites have also been prepared [73]. The conductivity of the whisker composite is larger than that of the PANI, although the corresponding to the nanowires is lower. However, the Seebeck coefficient of the nanowire structure is larger than the other two at high temperature. This possibly results from the quantum confinement in the nanowire and an increase in the density of states per unit volume in the nanocomposite [73]. 

More recently, some efforts have been reported by enhancing the thermoelectric performance of a polymer matrix with bismuth telluride (Bi_2_Te_3_). By mixing Bi_2_Te_3_ as a powder with PEDOT:PSS results in a 2–3 fold increase of the power factor [74] indicating a promising route of making materials to be used for printing TE devices on flexible substrates. Nanorods of Bi_2_Te_3_ coated by PANI were created in order to generate a more ordered molecular arrangement of PANI. Thus, the electrical conductivity and thermoelectric power were enhanced with *ZT* = 0.0043 at room temperature [75]. PEDOT:PSS/Bi2Te3 composite films depict a maximum electrical conductivity of 421 S·cm^−^^1^ with 10 wt% Bi_2_Te_3_ corresponding to a *PF* = 9.9 µW·m^−^^1^·K^−^^2^ and *ZT* = 0.04 [76]. 

Other procedure to enhance the TE efficiency is by adding additives. So, flexible films of PEDOT:PSS and poly(acrylic acid) (PAA) with an additive reach values of *ZT* = 0.2 for a Bi2Te3 concentration of 95 wt% [77]. Other inorganic particle different from Bi2Te3 is tellurium. The power factor of PEDOT:PSS can be optimized by dispersing Te nanowires just changing the synthesis, the effect of nanowire morphology [78,79,80]. A conformationally organized PEDOT:PSS interfacial layer template by the Te nanowires produces a high conductivity and Seebeck coefficient, keeping the low thermal conductivity of the host polymer. A *ZT* as high as 0.1 has been obtained. 

Novel metal/polymer/metal structures present a new design to combine inorganic metals and organic polymers thus increasing the Seebeck coefficient up to 252 µV·K^−^^1^ on a Al/PEDOT:PSS/Al device [81]. Spherical gold nanoparticles rod-shaped (AuNR) have proven to be effective as increases the electrical conductivity (2000 S·cm^−^^1^) and decrease the Seebeck coefficient (12 µV·K^−^^1^)) with the increase of AuNR concentration in the PEDOT:PSS matrix [82]. 

Table 3, Table 4 and Table 5 summarize values of the main thermoelectric parameters in conductive polymers, polymer composites with carbons and inorganic materials, respectively. 

**Table 3:** Thermoelectric parameters of the most relevant conducting polymers at room temperature.

System	*σ*(S·cm^−^^1^)	* S* (µV·K^−^^1^)	*κ*(W·m^−^^1^·K^−^^1^)	PF (µW·m^−^^1^·K^−^^2^)	*ZT*	Refs.
PEDOT:Tos/ PEO-PPO-PEO electrochem. reduct.	∼1200	∼100		1270	∼1.02	[16]
PEDOT:PSS + EG treat.	∼980	∼70	0.23	469	0.4	[48]
PEDOS-C6 electrochem. reduct.	∼200	∼110		354.7		[83]
PEDOT:PSS + EG treat. + hydrazine reduct.	∼1300	∼49	0.3	320	0.3	[13]
PEDOT:Tos + TDEA reduct.	∼80	∼290	0.37	324	0.25	[13]
PEDOT:BTFMSI + hydrazine reduct.	∼1080	∼37	0.19	147	0.22	[10]
PEDOT:PSS electrochem. reduct.	∼25	∼90	0.17	23.5	0.041	[21]
PEDOT:PSS + DMSO 5%	298	12.65		4.78	∼0.001	[44]
PANI/CSA-doping in m-cresol	220	∼20		11		[25]
PANI doped with H_3_PO_4_	40	∼7		0.19		[17]
polyselenophene and its copolymers with 3-methylthiophene	0.1–54	20–98		2–12	0.034	[38]
Copolymer of 1,12-bis(carbazolyl) dodecane and thieno[3,2-b]thiophene and its copolymers with 3-methylthiophene	4 × 10^−^^5^−0.4	75–169		∼0.17–0.33		[52]
Phenylenevinylene block copolymers and their blends with MEH-PPV	6 × 10^−^^6^−14.4	7–531		∼10^−^^5^–1.33		[45]
PEDOT:PSS + Polythiophenes Bilayered nanofilms	125–200	11–17		∼1.5–6		[53]

**Table 4:** Thermoelectric parameters of the most relevant conducting polymers composites with carbon materials at room temperature.

System	*σ*(S·cm^−^^1^)	*S*(µV·K^−^^1^)	*κ*(W·m^−^^1^·K^−^^1^)	PF (µW·m^−^^1^·K^−^^2^)	ZT	Refs.
SWCNT/PEDOT:PSS, DMSO, GA	400	27	↑0.4	25	∼0.02	[56]
CNT/PEDOT stabilizer TCPP	980	70		500		[58]
CNT/PVAc	48	45	0.34		0.006	[55]
SWCNT/PEDOT:PSS PVAc	1000	41	0.2–0.4	160		[57]
SWCNT/PEDOT:PSS Layered structure	241	38.9		21.1		[84]
SWCNT/PANI	125	40		0.2	0.004	[59]
3D-CNT/PANI	40.35	23	0.29		0.0022	[59]
CNT-PANI nanofibers	15	10		0.16	0.0022	[85]
PANI coated CNT/PANI	28	21.6	0.4		0.001	[86]
poly(3-hexylthiophene) SWCNTs	1000	29		98		[61]
MWCNT/polithiophene	6	25	0.6		8.7 × 10^−^^4^	[87]
PANI/graphite composites	100	10	1.2	4.18	1.37 × 10^−^^3^	[63]
PANI/graphene nanosheets pellet	∼60	∼30		5.6		[64]
PANI/graphene nanosheets film	∼8	∼42		1.47		[64]
PANI/graphene nanoplatelets mechanical blending	123	34		14		[66]
PEDOT:PSS/expanded graphite	213	15		5.31		[68]
PEDOT:PSS/graphene fullerene	700	25	0.4		0.06	[69]

**Table 5:** Thermoelectric parameters of the most relevant conducting polymers composites with inorganic nanoparticles at room temperature.

System	*σ* (S·cm^−^^1^)	*S* (µV·K^−^^1^)		*κ* (W·m^−^^1^·K^−^^1^)		*PF* (µW·m^−^^1^·K^−^^2^)		*ZT*	Refs.
PEDOT:PSS PAA Bi_2_Te_3_	380	79	0.36		∼240		0.2		[77]
PEDOT:PSS Bi_2_Te_3_	250	150	0.558		131		0.08		[74]
PANI Bi_2_Te_3_ nanorods p-type	11.626	39	0.11		1.8		0.004		[74]
PANI Bi_2_Te_3_ nanorods n-type	23	−70			10				[74]
PEDOT:PSS Bi_2_Te_3_ films	421	18.6	0.07		9.9		0.04		[76]
PEDOT:PSS Te	19.3	163	0.22–0.3		70.9		0.1		[78]
PEDOT:PSS Te nanowire	∼15	260			100				[79]
PEDOT:PSS Te nanowire	∼12	170			35				[80]
PEDOT:PSS Gold nanorod	∼2000	12			20				[80]
PH3T Bi_2_Te_3_	∼4.5	118			6.3				[88]

## 4. Theoretical Models of Thermoelectric Transport in Polymers

From the phenomenological point of view, there are mainly two theoretical approaches to the transport properties of conducting polymers [89]. The approaches depend on the strength of the electron-phonon interaction. If the electron-phonon interaction is strong, the electrons can be completely localized and the hopping between the polymer chains dominate the transport. On the other side, if the electron-phonon interaction is weak, a band model can be used to describe the electronics, and the electron-phonon interaction can be introduced by means of perturbation theory. In between, we have the polaron model. A polaron is an electron dressed with phonons; electrons move with a larger effective mass or a renormalized mass due to the coupling with the phonons and, consequently, a smaller mobility. Actually, electronic transport can always be addressed by considering polarons. 

The polyaniline was discovered 150 years ago, but until the 80’s its high electrical conductivity was not measured. The polyaniline is the first intrinsically conducting polymer discovered. Most of the models explaining the conducting properties of the polyanyline were developed in the 90’s it is thus one of the best known ICPs. The models developed at this time were devoted to the analysis of the behaviour of *σ* (dc and ac) and *α* as a function of temperature, electric field, level of localization, etc. Basically, all the models are based on those previously developed to study electric transport in disordered semiconductors (amorphous semiconductors mainly). The thermal conductivity of polymers was addressed later using molecular dynamic (MD) techniques, which are based on classical mechanics. The main problem in the use of MD is to find a good interaction potential. 

In the last years, *ab initio* techniques have also been applied to polymers. The main studies were on electronic transport: *σ*, *α* [89] and more recently *κ_e_* [90]. If the polymer is crystallized, usual density functional theory can be applied. 

### 4.1. Phenomenological Models

Most of the phenomenological models used to study the electrical transport in polymers are based on the existence of variable-range hopping (VRH) between the polymer chains [91]. These models are valid at low temperatures and low electric fields, the electrical conductivity can be written as:

(11) $$\sigma =\sigma_{0}e^{\beta F^{1/2}}e^{{\left(-T_{0}/T\right)}^{1/(1+d)}}$$

where *σ*_0_ = *en_e_µ_e_*; *n_e_* being the carrier concentration and *µ_E_* the mobility; *β* a disorder parameter; *F* the electric field; *d* accounts for the dimensionality of the system and T0 is given by

(12) $$T_{0}=\frac{8}{lD(E_{F})k_{B}}$$

*D*(*E_F_*) being the density of electronic states at the Fermi level and *l* is the localization length. In the most common case, *d* = 1, the conduction is one dimensional (1D). In the basic approach, there is practically no difference in the results given by a 1D or a 3D (three dimensional) model [92]. The model for charge transport in conducting polymers, developed by Wang *et al.* [92], is based on the transport equations coupled with the Poisson equation. Since the final results are numerical, the model starts from the basic case (1D), analyzing only the temperature variation of the conductivity, then they compare with the 2D case, next they introduce the effect of the electric field and finally solve the complete equations in the 1D case, a simplification shown to be valid. In the framework of the VRH model, the Seebeck coefficient in a polymer has two contributions, one coming from interlayer hopping, which can be shown to be constant (*α*_0_), and that whose origin is the intralayer hopping, with a 1/*T* behaviour [93]:

(13) $$\alpha =\alpha_{0}+\frac{C}{T}$$

where *C* is a constant. Actually, the Seebeck coefficient has a little bit more complicated dependence, as has been shown by [91], it decreases at low temperature and increases at high temperatures if the dependence of the mobility on temperature is taken into account. 

In conducting polymers, as in semiconductors, the phonon contribution to the thermal conductivity is usually more important than the electron contribution, unless the doping level gives origin to a metallic state. Molecular dynamics is the usual technique to calculate the thermal conductivity in polymers and polymer nanocomposites. Desai *el al.* [94] have analysed the transport in nanocomposites using a Leonnard-Jones potential

(14) $$U(r)=\delta \left\{4\left[{\left(\frac{r_{0}}{r}\right)}^{1/2}-{\left(\frac{r_{0}}{r}\right)}^{1/6}\right]+1\right\}\;(r>2^{1/6}r_{0})$$

otherwise *U* = 0. Nanoparticles (filler) are modelled as spheres and several attractive and repulsive potentials are deduced by Desai *et al.* [94] for the two and three-dimensional cases. They conclude that the monomers chains remain unchanged with a moderate filler content, while the diffusivity is enhanced near a repulsive surface, while it is strongly reduced near an attractive surface. The combination of polymers (co-polymers), for instance polyaniline and polyacetylene [95], has a positive impact on the phonon thermal conductivity reduction. Non equilibrium molecular dynamics simulations show that the intrinsic coupling between longitudinal and transverse vibrational modes impedes longitudinal thermal transport [95], while electrical transport remains the same. 

### 4.2. Models Based on ab Initio Techniques

In the calculation of the thermal transport, MD plays a crucial role, but it has also the limitations inherent to the use of classical mechanics. For instance, it cannot reproduce the behavior of the thermal conductivity as a function of temperature since a quantum statistics should be included [96]. MD cannot deal with the transport in localized systems, where the role of individual phonons is important. When we reduce the length scale, ballistic transport (for electrons and phonons) are important. Clearly, an atomistic approach, based on Quantum Mechanics is needed to go beyond all these problems. 

In a recent review, Wang *et al.* [89] employed first principles band structure calculations to obtain the electronic band structure of pentacene thin films and rubrene. They used the VASP code [97] (Viena ab initio simulation package) and selected the projector-augmented wave method (PAW) [98] within the generalized gradient approximation (GGA) [99]. However, even if first principles are applied, a fundamental model to account for the electrical and thermal transport is needed. Wang *et al.* [89] started from the Boltzmann equation for electrons to obtain an expression for the electronic contribution to the electrical and thermal transport (see Introduction). Instead of the conventional procedure of using a constant relaxation time, they include the interaction of electrons with acoustic phonons calculating the shift of the bands produced by the dilation produced by the lattice displacement (deformation potential interaction [100]). The calculation of the transport coefficients (*σ*, *α* and *κ_e_*) are performed with the BoltzTrap package that interfaces with VASP. The lattice thermal conductivity is calculated with three MD packages, GAFF [101] (generalized amber force field), OPLS [102] and LAMMPS [103]. The calculations for the Seebeck coefficient as a function of the carrier density in two-dimensions [92] follows the experimental trend, decreasing linearly in an Arrhenius plot, but in the case of rubrene the experimental slope is much higher than the theoretical calculations. In the case of MD simulations, the inverse of the lattice thermal conductivity scales linearly with the inverse of the length [104], giving a similar value to that found experimentally. The figure of merit was also calculated as a function of the chemical potential or carrier concentration. In the case of pentacene, *ZT* ≈ 1.5, while for the rubrene the maximum *ZT* ≈ 0.6 [89]. A similar calculation is made for P3HT nanowires, giving a maximum value of *ZT* ≈ 1.5. 

There is another way to calculate the thermal properties also based on ab initio methods. The starting point are the Onsager relations and the Landauer theory of quantum transport [105]. The electrical conductivity, Seebeck coefficient and electronic thermal conductivity tensor can be written as [106]:

(15) *σ*(*µ*, *T*) = e^2^L_00_(*µ*, *T*)

(16) $$\alpha (\mu ,T)=\frac{1}{eT}L_{01}(\mu ,T)L_{00}^{-1}(\mu ,T)$$

(17) $$\kappa_{e}=\;\frac{1\;}{T}\left[L_{11}(µ,\;T)+L_{01}(µ,\;T)L_{00}^{-1}(µ,\;T)L_{01}(µ,\;T)\right]$$

where *µ* is the chemical potential (*µ* = *E_F_* at *T* = 0) and

(18) $$L_{mn}=-\frac{1}{A}\int_{-\infty }^{+\infty }d\varepsilon T_{e}(\varepsilon ){\left(\varepsilon -µ\right)}^{m+n}\frac{\partial f(\varepsilon ,\;µ,\;T)}{\partial \varepsilon }$$

*T_e_* being the electron transmission function. The derivative of the electron distribution function *f* is peaked around *µ*. Similarly, the phonon lattice conductivity can be given by

(19) $$\kappa_{p}(T)=\frac{1}{A}\int_{0}^{\infty }d\omega T_{p}(\omega )\hbar \omega \frac{\partial n(\omega ,\;T)}{\partial T}$$

The transmission functions *T_e_*(*ε*) and *T_p_*(*ω*) can be given in terms of the electron band structure and the phonon dispersion, respectively:

(20) $$T_{e}=\frac{1}{N}\sum_{n,k}\tau_{n,k}v(n,k)⊗v(n,k)D_{e}(\varepsilon )\;\text{and }T_{p}=\frac{1}{N}\sum_{\nu ,q}\tau_{\nu ,q}v(\nu ,q)⊗v(\nu ,q)D_{p}(\omega )$$

which can also be expressed in terms of the retarded Green functions [90] in the case of electronic transmission. In this formalism, electrons and phonons are treated ballistically. The Kapitza resistance (thermal resistance of the interface) has been recently calculated by using the Landauer formalism [107]. A similar approach is followed by Calzolari *et al.* [108]. They calculate the electronic contribution using the Landauer formalism with the Green function method and the lattice dynamics using the package Quantum Expresso (http://www.quantum-espresso.org/) through the calculation of the dynamical matrix. Then, they implemented the calculation of the thermal transport into the WANT package (http://www.wannier-transport.org/) based on Wannier functions. The model is applied to the calculation of the thermal properties of crystalline polyethylene [109]. 

In a recent work, Bao *et al.* [110] calculate, also based on the Landauer formalism, the electrical conductivity of carbon nanotube (CNT) nanocomposites. They created a percolation network using a Monte Carlo simulation and then the tunneling between the nanotubes. They show that the single wall CNTs plays a predominant role as compare with the multi-wall CNTs in conduction. 

## 5. Summary

In this work, we have reviewed the progress of the transport properties of conductive polymers and polymer composites in the last 20 years under the scope of thermoelectric applications. While inorganic materials, in spite of growing them in form of nanostructures, have improved the figure of merit a factor of 3 in the last 20 years, the figure of merit of organic materials have been improved several orders of magnitude. We believe that there is still room for improvement. During the last two years the evolution of the figure of merit *ZT* has reached values of 0.2 − 0.4. The different routes drawn up in very recent works allows us to envisage a *ZT* ∼ 1 within one year from now. 

From the theoretical point of view, there are significative advances in the prediction of the transport properties of crystalline polymers using *ab initio* techniques or molecular dynamics in combination with *ab initio* models. A joining effort from experimentalists and theoreticians is needed to further improve the understanding of transport in polymers and as an output, to compete with inorganic materials not only because of the classical constrains, but because the efficiency of polymer-based TEG overcome that of inorganic. 

## Abbreviation List

PEDOT: poly(3,4-ethylenedioxythiophene)
PPy: polypyrrole
PANI: polyaniline
Tos: p-toluenesulfonate
PSS: polystyrenesulfonate
MEH-PPV: poly-(2-methoxy-5-(2-ethylhexyloxy)phenylenevinylene)
CNT: carbon nanotube
GN: grafene
TCPP: meso-tetra(4-carboxyphenyl) porphine
PEDOS-C6: poly(3,4-ethylenedioxyselenophene)
SWCNT: singlewall carbon nanotube
DWCNT: doublewall carbon nanotube
MWCNT: multiwall carbon nanotube
P3HT: poly(3-hexylthiophene)
Pth: polythiophene
P3MeT: poly(3-Metylthiophene)
PEO-PPO-PEO: poly(ethylene glycol)-block-poly(propylene glycol)-blockpoly(ethylene glycol) triblock copolymer
CSA: camphorsulfonic acid
EG: ethylene glycol
DMSO: dimethylsulfoxide
PVA: polyvinyl alcohol
PEI: polyethyleneimine
PVAc: poly(vinyl acetate)
PAA: poly(acrylic acid)
BTFMSI: bis(trifluoromethylsulfonyl)imide
TDEA: tetrakis(dimethylamino)ethylene
